# The association between “Brain‐Age Score” (BAS) and traditional neuropsychological screening tools in Alzheimer's disease

**DOI:** 10.1002/brb3.1020

**Published:** 2018-06-22

**Authors:** Iman Beheshti, Norihide Maikusa, Hiroshi Matsuda

**Affiliations:** ^1^ Integrative Brain Imaging Center National Center of Neurology and Psychiatry Kodaira Tokyo Japan

**Keywords:** ADAS, Alzheimer's disease, anatomical MRI measurements, brain‐age score, CDR, FAQ, mild cognitive impairment, MMSE, neuropsychological screening, structural MRI

## Abstract

**Introduction:**

We present the Brain‐Age Score (BAS) as a magnetic resonance imaging (MRI)‐based index for Alzheimer's disease (AD). We developed a fully automated framework for estimating the BAS in healthy controls (HCs) and individuals with mild cognitive impairment (MCI) or AD, using MRI scans.

**Methods:**

We trained the proposed framework using 385 HCs from the IXI and OASIS datasets and evaluated 146 HCs, 102 stable‐MCI (sMCI), 112 progressive‐MCI (pMCI), and 147 AD patients from the J‐ADNI dataset. We used a correlation test to determine the association between the BAS and four traditional screening tools of AD: the Mini‐Mental State Examination (MMSE), Clinical Dementia Ratio (CDR), Alzheimer's Disease Assessment Score (ADAS), and Functional Assessment Questionnaire (FAQ). Furthermore, we assessed the association between BAS and anatomical MRI measurements: the normalized gray matter (nGM), normalized white matter (nWM), normalized cerebrospinal fluid (nCSF), mean cortical thickness as well as hippocampus volume.

**Results:**

The correlation results demonstrated that the BAS is in line with traditional screening tools of AD (i.e., the MMSE, CDR, ADAS, and FAQ scores) as well as anatomical MRI measurements (i.e., nGM, nCSF, mean cortical thickness, and hippocampus volume).

**Discussion:**

The BAS may be useful for diagnosing the brain atrophy level and can be a reliable automated index for clinical applications and neuropsychological screening tools.

**Highlights**

- We present the Brain‐Age Score (BAS) as a MRI‐based index for AD.
- Our automated computer‐aided framework estimates the BAS in healthy and MCI/AD brains.
- The BAS is in line with traditional screening tools of AD as well as anatomical MRI measurements.
- The BAS could be used for diagnosing brain atrophy as a reliable automated index.

## 1 INTRODUCTION

Assessments of Alzheimer's disease (AD) have relied almost extensively on cognitive and functional assessments such as the Mini‐Mental State Examination (MMSE) (Folstein, Folstein, & McHugh, 1975), the Clinical Dementia Rating (CDR) (Morris, 1993), the Alzheimer's Disease Assessment Score (ADAS) (Mohs, Rosen, & Davis, 1983), and the Functional Assessment Questionnaire (FAQ) (Fillenbaum & Smyer, 1981), plus a complete review of the medical history of the subject and his/her family members. These traditional screening tools are generally performed based on simple surveys that has been shown to be associated with common method bias (Presser et al., 2004). These methods also suffer from a lack of certainty due to factors such as the patient's mood, the existence of other important events or illnesses, side effects from other treatments, and the patient's level of education. Thus, bias in these survey methods increases the uncertainty in the diagnosis of AD.

With respect to this point, an automated and physician‐friendly computer screening of AD is urgently needed. Several research groups have investigated various fully automated approaches in AD studies (Bedell et al., 2014; Beheshti, Olya, & Demirel, 2016; Cuingnet et al., 2011; Franke, Ziegler, Klöppel, & Gaser, 2010; J Martinez‐Murcia, Górriz, Ram'irez, & Ortiz, 2016; Klöppel et al., 2008; Mikhno, Redei, Mann, & Parsey, 2015). Despite recent progress in the automated assessment of AD, the development of an automatic approach that addresses the uncertainty of diagnosing AD is still challenging and requires further data. Previous studies have shown that neuroimaging data along with advanced pattern recognition techniques can be used for predicting clinical scores (Moradi, Hallikainen, Hänninen, Tohka, & Neuroimaging, 2017; Shen et al., 2011; Stonnington et al., 2010) as well as chronological age (Cole, 2017).

The brain‐age technique was recently introduced as a powerful biomarker that can be used to estimate an individual's neuroanatomical age (Duchesne & Gravel, 2016; Franke et al., 2010). The use of the brain‐age technique has helped reveal the abnormal brain changes in many brain studies such as those of AD (Franke et al., 2010), the prediction of the conversion of mild cognitive impairment (MCI) to AD (Gaser, Franke, Klöppel, Koutsouleris, & Sauer, 2013), and investigations of the brains of children and adolescents (Franke, Luders, May, Wilke, & Gaser, 2012), long‐term meditation practitioners (Luders, Cherbuin, & Gaser, 2016), and schizophrenia patients (Koutsouleris et al., 2014).

Here, we introduce the “Brain‐Age Score” (BAS), which we define as the difference between an individual's chronological age and his or her neuroanatomical age, as a fully automated and reliable index that can be used to assess the level of AD in individual subjects. We developed an automated brain‐age framework based on voxel‐based morphometry (VBM) features obtained from structural magnetic resonance imaging (sMRI) data in order to estimate the neurological age among healthy controls, individuals with MCI, and individuals with AD.

The proposed framework was trained on 385 samples from the IXI dataset (http://www.brain-development.org/ixi-dataset/) and the OASIS dataset (http://www.oasis-brains.org/). We evaluated the proposed framework on 147 AD patients, 112 progressive‐MCI (pMCI) patients, 102 stable‐MCI (sMCI) patients, and 146 healthy controls (HCs) acquired from the J‐ADNI dataset. More information about J‐ADNI is provided in the Appendix and (Fujishima et al., 2017). To test the efficiency of the proposed framework, we performed a correlation test between the BAS and the neuropsychological screening tools (i.e., MMSE, CDR, ADAS, and FAQ) as well as between the BAS and anatomical MRI measurements (i.e., normalized gray matter (nGM), normalized white matter (nWM), normalized cerebrospinal fluid (nCSF), mean cortical thickness, and hippocampus volume).

In light of the relevant literature, we hypothesized that the BAS would show adequate performance in the screening of the level of AD in a fully automated manner, using only a standard MRI scan. Our findings demonstrate that (1) the BAS is capable of being used to diagnosis an individual's level of brain atrophy, and (2) it can be considered a reliable automated index for clinical applications as well as neuropsychological screening tools.

## 2 LITERATE REVIEW

In this section, we review the brain‐age models as well as feature selection procedures in a series of neuroimaging studies. Several research groups have investigated automated methods to estimate the neuroanatomical age from sMRI data. As an example, the team of Gaser and colleagues (Franke et al., 2010) introduced an automated framework in the basis of GM density maps through a standard VBM procedure and a regression model for estimating the brain‐age of healthy subjects. They examined the influence of different factors such as MRI preprocessing, regression models, data reduction, the use of different scanners, and the training sample size on the age estimation accuracy using sMRI data. In another study (Gaser et al., 2013), the researchers modeled a brain‐age framework using a structural MRI database of 320 healthy controls obtained from the IXI and OASIS datasets, and they estimated the individual brain ages of MCI patients from the ADNI dataset. They reported an accuracy value of up to 81% for predicting conversion to AD in MCI patients at the baseline. The authors in (Franke & Gaser, 2012) presented longitudinal alterations in BAS in the 150 AD patients, 112 pMCI patients, 36 sMCI patients, and 108 HCs from the ADNI dataset, where correlation values of *r* = −0.46, *r* = 0.39 and *r* = 0.45 (*p *<* *0.001) were achieved between baseline BAS and the MMSE, the CDR and the ADAS scores, respectively. In (Koutsouleris et al., 2014), the researchers examined neuroanatomical age estimation in individuals with schizophrenia and other mental disorders. They trained their proposed brain‐age framework using a structural MRI database of 800 healthy subjects examined at five different centers, and then, they evaluated individuals in at‐risk mental states for psychosis, borderline personality disorder, and major depression and schizophrenia subjects. The researchers in (Luders et al., 2016) considered a brain‐age framework to investigate the neuroanatomical age in subjects who had been regularly engaging in meditation for a period of years. They examined over 650 subjects in the training phase and then evaluated a large sample of the same type of meditating subjects. They observed that at age 50, the brains of the meditating subjects were younger than those of age‐matched healthy controls. In (Cole, Leech, & Sharp, 2015), the authors built a brain‐age model in basis of GM and WM density maps on 1,537 healthy individual and then tested on 113 independent healthy controls and 99 patients after traumatic brain injury (TBL). They stated a mean BAS of 4.66 and 5.97 years for GM and WM modalities, respectively, among TBL patients. In another study (Cole et al., 2017), the authors conducted a brain‐age model by combining the GM and WM modalities from 2,001 healthy controls to investigate the brain age on HIV‐positive and HIV‐negative subjects. According to this research, they realized that HIV‐positive subjects have a significantly greater BAS in comparison with HIV‐negative subjects.

Many researchers have presented different dimensionality reduction and feature selection methods in machine learning for neuroimaging studies. For instance, the authors in (López et al., 2009) conducted a data reduction on features extracted from single photon emission computed tomography (SPECT) and positron emission tomography (PET) images by means of principal component analysis (PCA). In (Ramírez et al., 2010) a new data reduction method was introduced by the means of partial least squares (PLSs) to overcome the curse of dimensionality. The authors applied the proposed PLS‐based method on SPECT data and extracted the score features for an AD classification task. The PLS‐based data reduction has been widely used in different neuroimaging studies (Chaves, Ramírez, Górriz, & Puntonet, 2012; Khedher, Ramírez, Górriz, Brahim, & Segovia, 2015; Ramírez et al., 2010; Segovia, Górriz, Ramírez, Salas‐González, & Álvarez, 2013). The researchers in (Liu, Zhang, & Shen, 2016) used a sparse‐based feature selection method to find informative features from template space for AD classification and MCI conversion prediction. In (Beheshti & Demirel, 2016), the authors proposed a feature‐ranking strategy for identifying the most informative features from a high‐dimensional space in an AD classification task. The dimensionality of selected features was determined by the mean of fisher criterion. It is worth nothing that the most of these approaches were hired in classification studies. In this study, we present an automatic feature selection approach in the basis of feature‐ranking strategy for brain‐age framework.

## 3 STUDY PARTICIPANTS

A total of 892 structural MRI scans from the IXI, OASIS, and J‐ADNI datasets were used. To separate the data used for training from the data used for evaluating, we divided the dataset into two main groups: (1) Training data: 385 healthy samples from the IXI and OASIS datasets of subjects aged ≥50 years, and (2) Evaluating data: 507 samples from the J‐ADNI database. We divided the total of 507 participants into four groups based on criteria as follows:

- AD subjects: MMSE score of 20–26, CDR 0.5 or 1, and memory complaint.
- HC subjects: MMSE score 24–30, CDR of 0, non‐depressed, no memory complaint.
- sMCI subjects: MMSE score 24–30, memory complaint (preferably corroborated by an informant), objective memory loss measured, a CDR of 0.5, absence of significant levels of impairment in other cognitive domains, and essentially preserved activities of daily living (if the diagnosis was MCI for ≥36 months).
- pMCI subjects: MMSE score 24–30, memory complaint (preferably corroborated by an informant), objective memory loss measured, a CDR of 0.5, absence of significant levels of impairment in other cognitive domains, essentially preserved activities of daily living (if the diagnosis was MCI at baseline but conversion to AD was reported after baseline within 6–36 months).

All of the subjects whose samples were evaluated had taken the MMSE, CDR, ADAS, and FAQ as neuropsychological screening tools. The evaluating data were acquired at the baseline. Table 1 summarizes the demographic and clinical characteristics of the subjects. This study was approved by the Institutional Review Board at the National Center of Neurology and Psychiatry, Tokyo, Japan.

**Table 1:** Characteristics of the study participants

Training data (*n* = 385)			Evaluating data (*n* = 507)^a^			
Dataset:	IXI	OASIS	J‐ADNI	J‐ADNI	J‐ADNI	J‐ADNI
Category	HC	HC	HC	sMCI	pMCI	AD
No. of subjects	274	111	146	102	112	147
Females/males	170/104	82/29	78/68	57/45	65/47	84/63
Age (yrs)	63.50 [7.64]	70.86 [10.68]	68.28 [5.61]	73.44 [5.97]	73.62 [5.57]	74.07 [6.57]
MMSE	n/a	29.07 [1.17]	29.11 [1.20]	26.55 [1.81]	26.08 [1.53]	22.55 [1.80]
CDR	n/a	0.00 [0.00]	0.00 [0.00]	0.50 [0.00]	0.50 [0.00]	0.67 [0.23]
ADAS	n/a	n/a	7.40 [4.24]	17.85 [6.08]	22.20 [5.78]	27.42 [5.84]
FAQ	n/a	n/a	0.12 [1.15]	2.51 [2.88]	4.64 [4.78]	10.16 [5.90]

*Notes*

All data are mean [std. dev.] mode.

AD: people with Alzheimer's disease; CDR: clinical dementia rating; HC: healthy control participants; sMCI: stable mild cognitive impairment; pMCI: progressive mild cognitive impairment; MMSE: mini‐mental state examination; FAQ: functional assessment questionnaire; ADAS: alzheimer's disease assessment score; n/a: not available.

a Only data at the baseline were used in the evaluation.

## 4 METHODS

**Figure 1:**
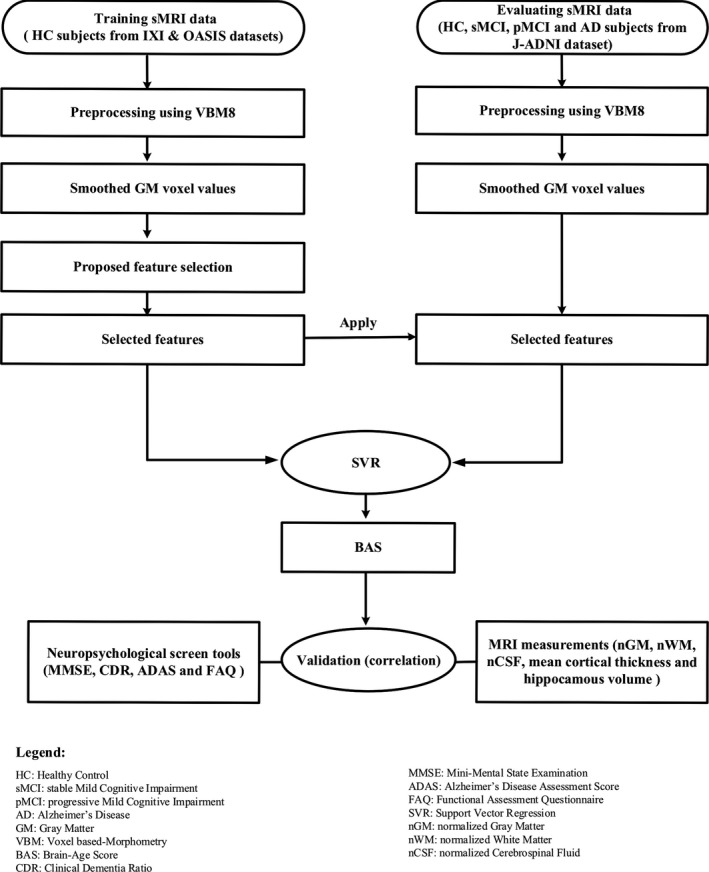
The pipeline of the proposed framework for estimating the BAS (Brain‐Age Score)

Here, we describe the computational processes that we applied to the data. The pipeline of the proposed framework is illustrated in Figure 1; the protocol was as follows. (1) We performed MRI preprocessing using a VBM approach on T1‐weighted MRI data. (2) We used our proposed automated feature selection approach to select the most informative features. (3) We trained the proposed framework using the training data and then assessed the framework using the evaluating data. (4) We tested the results of our proposed approach using a correlation test between the BAS values and neuropsychological subjects’ scores (i.e., MMSE, CDR, ADAS, and FAQ) as well as the subjects’ anatomical MRI measurements (i.e., nGM, nWM, nCSF, mean cortical thickness, and normalized hippocampus volume). Detailed explanations concerning the above‐described steps are provided next.

### 4.1 MRI preprocessing

All T1‐weighted MRI scans were preprocessed using the SPM8 package (http://www.fil.ion.ucl.ac.uk/spm/software/spm8/) and the standard VBM8 toolbox (http://dbm.neuro.uni-jena.de/vbm). With the VBM8 toolbox, all of the subjects’ MRI scans were bias‐corrected, special normalized and decomposed into the gray matter (GM), white matter (WM), and cerebrospinal fluid (CSF) components. In this study, we used only the GM images. As proposed in study (Franke et al., 2010), we processed the GM images using an affine registration and then spatially smoothed with an 8‐mm full‐width‐half‐maximum Gaussian kernel followed by spatial resolution to 8 mm. The voxel values for each subject were considered the MRI features.

To evaluate the association between BAS values and anatomical MRI measurements, we performed a FreeSurfer image analysis ver. 5.3.0. (https://surfer.nmr.mgh.harvard.edu/) on evaluating data to extract the hippocampus volumes as well as cortical thickness values. For each subject, the left and right hippocampus volumes were calculated and added together. The mean cortical thickness was computed by averaging of whole‐brain cortical thickness values. Besides, the whole‐brain GM, WM, and CSF volumes were extracted through VBM analysis. We normalized the GM, WM, and CSF volumes as well as hippocampus measurements for head size by dividing the respective intracranial volumes and considered as nGM, nWM, nCSF, and normalized hippocampus volumes, respectively.

### 4.2 Proposed feature selection

The purpose of feature selection is to identify the most informative subset of the available features not only for data reduction, but also for improving the performance. In this context, we present a new and automatic feature selection approach based on feature‐ranking for our proposed brain‐age framework. The purpose of any feature‐ranking strategy is to sort the features based on their information and then select an optimal informative subset in order to speed up the learning process and promote the performance of models (Zhou & Wang, 2007). The details of the feature‐ranking strategy were as described (Beheshti & Demirel, 2016; Beheshti, Demirel, Farokhian, Yang, & Matsuda, 2016). The proposed feature selection approach is applied only on training data. In the present study, we sorted all voxels based on the respective correlation value of each voxel value and the subjects’ chronological age, in an ascending order. Then, we increased the number of ranked features from 1 to utmost number of features, along with the calculation of the respective mean absolute error (MAE) during the cross‐validation strategy. Based on the cross‐validation strategy, we divided the training data into different *k*‐folds, where, in each step, *k*‐1 folds are randomly used for training a regression model and the remained fold for testing. This process is performed *k* different times by leaving a different fold as test data, which are then used to estimate the respective *MSE* values. In this study, *k *=* *5 was used. For each iteration (i.e., increasing the number of ranked features), the total MAE was calculated by the averaging of MAEs obtained across the cross‐validation.

The optimal number of ranked features that minimizes the total MAE was selected as the optimal subset. Algorithm 1 shows the pseudo‐code of the proposed feature selection method to determine the optimal‐feature‐subset. The advantage of this procedure is that the top informative voxels are determined in a fully automated manner.

Algorithm 1 The pseudo‐code of the proposed feature selection method.

**Table:** 

** ● V** ← component_set (Data_Train_, Age_Train_) **●** Ranked features ← correlation ranking method (**V**) **●** number of top ranked features← Ø **●** Ψ = 3,747% number of features **●** *k* = 5 **● for ** *n* = 1 to Ψ **do**
**R** ← Ranked features (1: *n*, Age_Train_)
Total_MAE (*n*) ← ∞
Cross‐validation (Set **R**) {
Repeat *k* times
Randomly divide **R** into *k*‐folds, *F* = {f_1_, f_2_, …, f_k_}
**Foreach** fold f_i_ {
MAE_i_ ← ∞,
S_train_ ← *F* – {f_i_}
S_test_ ← f_i_
Train the SVR on S_train_
Predicted age ← Test the SVR on S_test_
calculate the MAE_i_ } }
Total_MAE (*n*) ← mean MAE_i_ **● end for** **●** number of top ranked features ← arg min *Total_MAE* )*n* ∊ 1, …, ψ **● Return** Ranked features (1, 1: number of top ranked features)

### 4.3 The support vector regression algorithm

For the prediction of the brain age, we established the framework using a support vector regression (SVR) algorithm (Smola & Schölkopf, 2004) because of its desirable characteristics and easy computation for a high‐dimensional feature space. The estimation model in the basis of SVR algorithm has been widely used in different neuroimaging studies (Dosenbach et al., 2011; Erus et al., 2015; Koutsouleris et al., 2014; Lancaster et al., 2018).In the present study, a linear v‐support vector regression (v‐SVR) performed using LIBSVM (http://www.csie.ntu.edu.tw/~cjlin/libsvm/) toolbox with a default setting (i.e., in the LIBSVM: *C* = 1, *v *=* *0.5) was used. The linear regression function *f* (*x*) can be represented as follows (Zhang, Zheng, Xia, Wang, & Chen, 2017).

(1) $$f\left(x\right)=w\cdot \varphi \left(x\right)+b$$

in which *x* stands for an input space (i.e., the MRI features in our study) and φ refers to a kernel function. Besides, *b* and *w* denote the offset for the regression line and the slope, respectively.

More information about the theoretical background of the SVR can be found in (Scholkopf et al., 1997; Smola & Schölkopf, 2004; Vapnik, 2013; Welling, 2004).

### 4.4 Validation and statistical analysis

We calculated the accuracy of the age estimator using the MAE and root mean square error (RMSE) as follows:

(2) $$\text{MAE}=[1/n*\sum_{i}|g_{i}^{\prime }-g_{i}]$$

(3) $$\text{RMSE}={\left[1/n*\sum_{i}|{\left(g_{i}^{\prime }-g_{i}\right)}^{2}\right]}^{1/2}$$

where *n* is the number of subjects in the test sample, and $g_{i}^{\prime }$ and $g_{i}$ denote the estimated age and the chronological age, respectively.

To test whether the BAS results are in line with the results of the four neuropsychological screening tools and hippocampus volume, we used the Pearson correlation test for the MMSE, ADAS, and FAQ screening tools as well as hippocampus volume and the Spearman correlation test for the CDR assessment. The statistical comparisons of groups were accomplished by an analysis of variance (ANOVA) followed by Tukey's multiple comparison tests, using the software program SPSS (Statistical Package for Social Sciences) (http://www.spss.com/). Probability values <0.05 were accepted as significant.

## 5 RESULTS

In this section, we evaluate the proposed framework on the J‐ADNI dataset including 147 AD, 112 pMCI, and 102 sMCI patients and 146 HCs for the determination of the association between the BAS and the neuropsychological screening tools as well as between the BAS and anatomical MRI measurements. All data used in the evaluation step were acquired at the baseline.

### 5.1 Performance of the proposed feature selection

**Figure 2:**
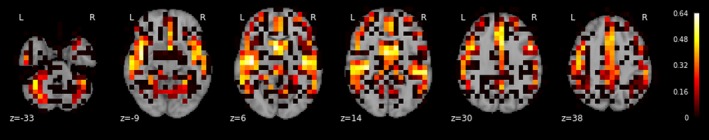
The illustration of selected voxels based on the absolute correlation of each voxel value and the subjects’ chronological age

As described above in section 4, the normalized and smoothing GM images were resampled to 8‐mm isotropic spatial resolution. This procedure generated 3,747 voxel values per subject, which were used as MRI features. We applied the proposed feature selection approach to select the most informative voxels for the brain‐age estimation. A total of 665 voxels were selected through proposed feature selection procedure. Figure 2 illustrates the selected voxels through proposed feature selection procedure. For the evaluation of the proposed feature selection procedure, we used the HCs from the J‐ADNI dataset.

The proposed feature selection method not only introduced a dimensionality reduction but also reduced the overall MAE from 4.35 with 95% confidence intervals [3.83–4.87] to 4.02 years with an overall correlation *r* = 0.68, *p *<* *0.001. Table 2 presents the details of the performance of the brain‐age framework using all feature vectors and our proposed feature selection approach as well as comparison to other studies. As can be seen from Table 2, our performance accuracy in terms of the MAE (4.02 years for the HC subjects) is quite comparable to other recent brain‐age estimations of healthy control subjects (Franke & Gaser, 2014; Franke et al., 2010; Gaser et al., 2013; Koutsouleris et al., 2014; Lin et al., 2016; Wang, Dai, Li, Hua, & He, 2012).

**Table 2:** The performance comparison of the proposed feature selection method with using all features and the state‐of‐the‐art techniques applied to HC subjects from the J‐ADNI dataset

	No. of features	MAE	RMSE
Franke et al., 2010	410^a^	4.98	6.28
Franke & Gaser, 2014	–	5.10	–
Koutsouleris et al., 2014	400^a^	4.60	–
Wang et al., 2012	–	4.60	5.60
Gaser et al., 2013	–	3.80	–
Lin et al., 2016	720	4.29	5.09
Using all features	3747	4.35	5.41
Proposed method	665	4.02	5.10

*Notes*

MAE: mean absolute error; RMSE: root mean square error.

a The number of principal components per subject.

### 5.2 Estimating the brain age in the evaluating group

We applied the proposed brain‐age estimation framework to the HCs and sMCI, pMCI, and AD patients from the J‐ADNI dataset to estimate the respective BAS values. A positive BAS indicates that the individual's brain is estimated to be older than his or her chronological age and that consequently, accelerated brain aging has occurred for this individual. Conversely, a negative BAS indicates that the individual's brain is estimated to be younger than his or her chronological age. As an example, when a 65‐year‐old individual exhibits a BAS of +5, he or she shows the typical aging pattern of a 70‐year‐old individual. In the same 65‐year‐old individual, a BAS of −5 indicates the typical aging pattern of a 60‐year‐old individual.

**Figure 3:**
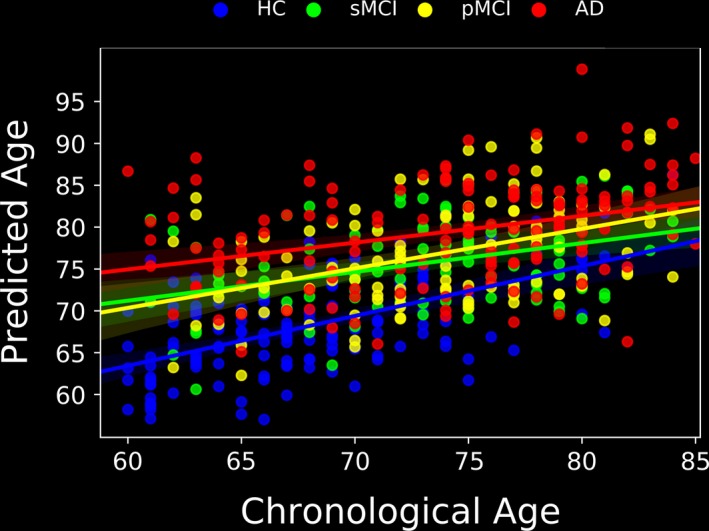
Scatterplot and linear fit for chronological age versus predicted age in the evaluation group (HC,* n* = 147; sMCI,* n* = 102; pMCI,* n* = 112; and AD,* n* = 146)

**Figure 4:**
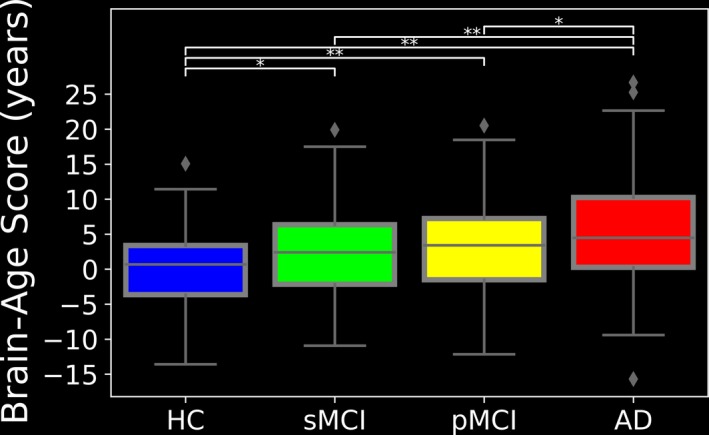
The box plots with the BAS (years) in the HC, sMCI, pMCI, and AD subjects from the J‐ADNI dataset. *p < 0.05, **p < 0.001

Figure 3 illustrates the chronological ages versus predicted ages for the entire evaluation group. Figure 4 provides the box plots with BAS (in years) for the four subject groups. The brain ages differed significantly across the subject groups (*F* [3,503] = 17.24, *p *<* *0.001). The mean BAS values were as follows: HCs: +0.07 year, sMCI patients: +2.38 years, pMCI patients: +3.15 years, and AD patients: +5.36 years. As can be seen from Figures 3 and 4, our proposed brain‐age estimator indicated differences in the BAS at different stages of AD, and our results suggest that across the spectrum of health/disease from healthy controls to individuals with sMCI, pMCI or AD, the mean of the BAS increases.

### 5.3 Association between the BAS and the traditional neuropsychological screening tools as well as between the BAS and anatomical MRI measurements

**Figure 5:**
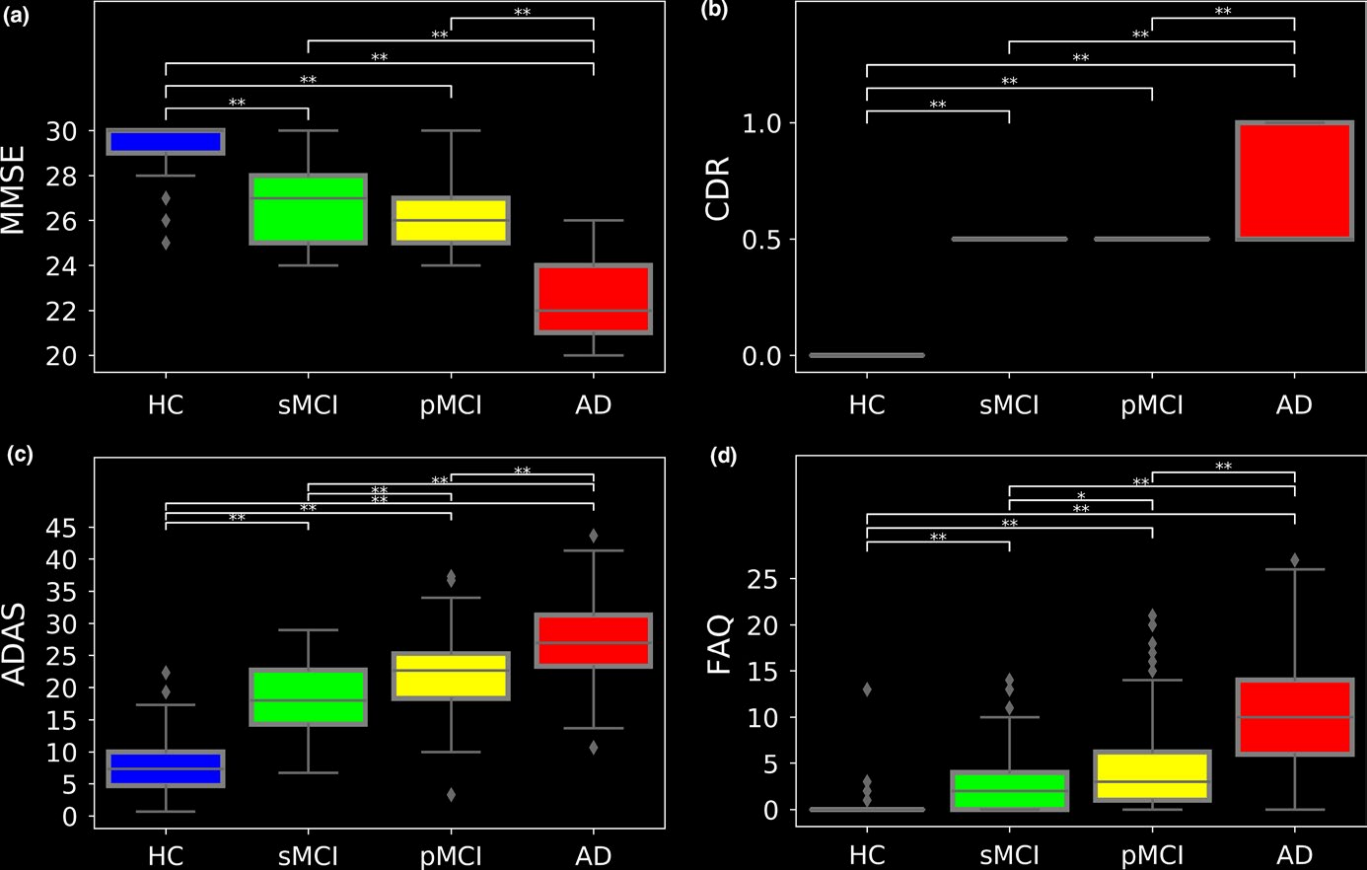
Box plots of neuropsychological score distributions for the evaluating group: (a) MMSE, (b) CDR, (c) ADAS, and (d) FAQ. The neuropsychological scores and hippocampus volumes were acquired at the baseline. *p < 0.05; **p < 0.001

**Figure 6:**
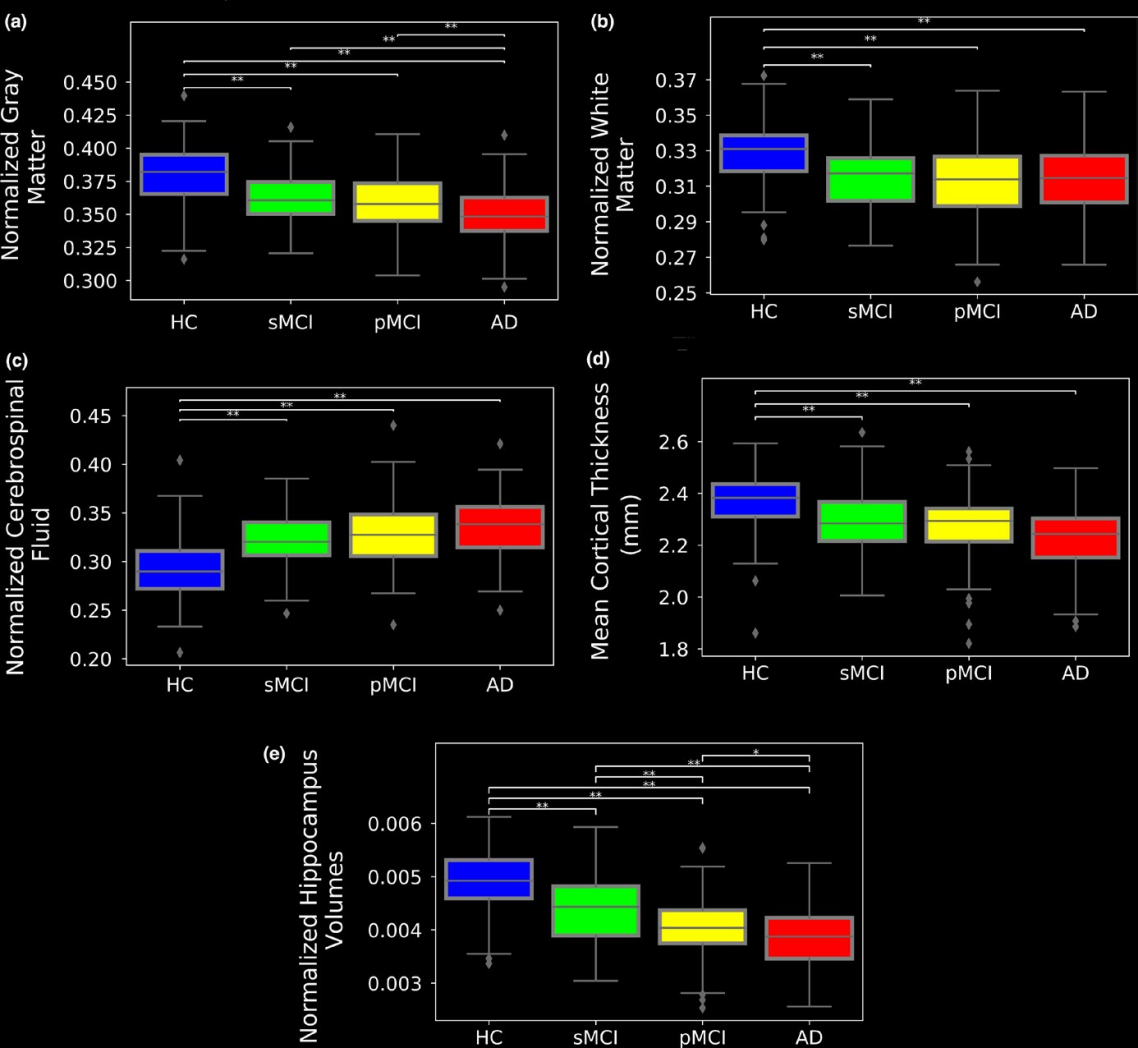
Box plots of anatomical MRI measurement distributions for the evaluating group: (a) nGM, (b) nWM, (c) nCSF, (d) mean cortical thickness, and (e) normalized hippocampus volume. The anatomical MRI measurements were acquired at the baseline. *p < 0.05; **p < 0.001

To determine the association between the BAS results and those of the four traditional neuropsychological screening tools as well as between the BAS results and anatomical MRI measurements, we analyzed the respective neuropsychological screening scores as well as anatomical MRI measurements in the 147 AD patients, 112 pMCI patients, 102 sMCI patients, and 146 HCs from the J‐ADNI dataset. The results of the neuropsychological subjects’ scores (i.e., MMSE, CDR, ADAS, and FAQ) as well as the subjects’ anatomical MRI measurements (i.e., nGM, nWM, nCSF, mean cortical thickness, and normalized hippocampus volume) are given in Figures 5 and 6, respectively.

**Figure 7:**
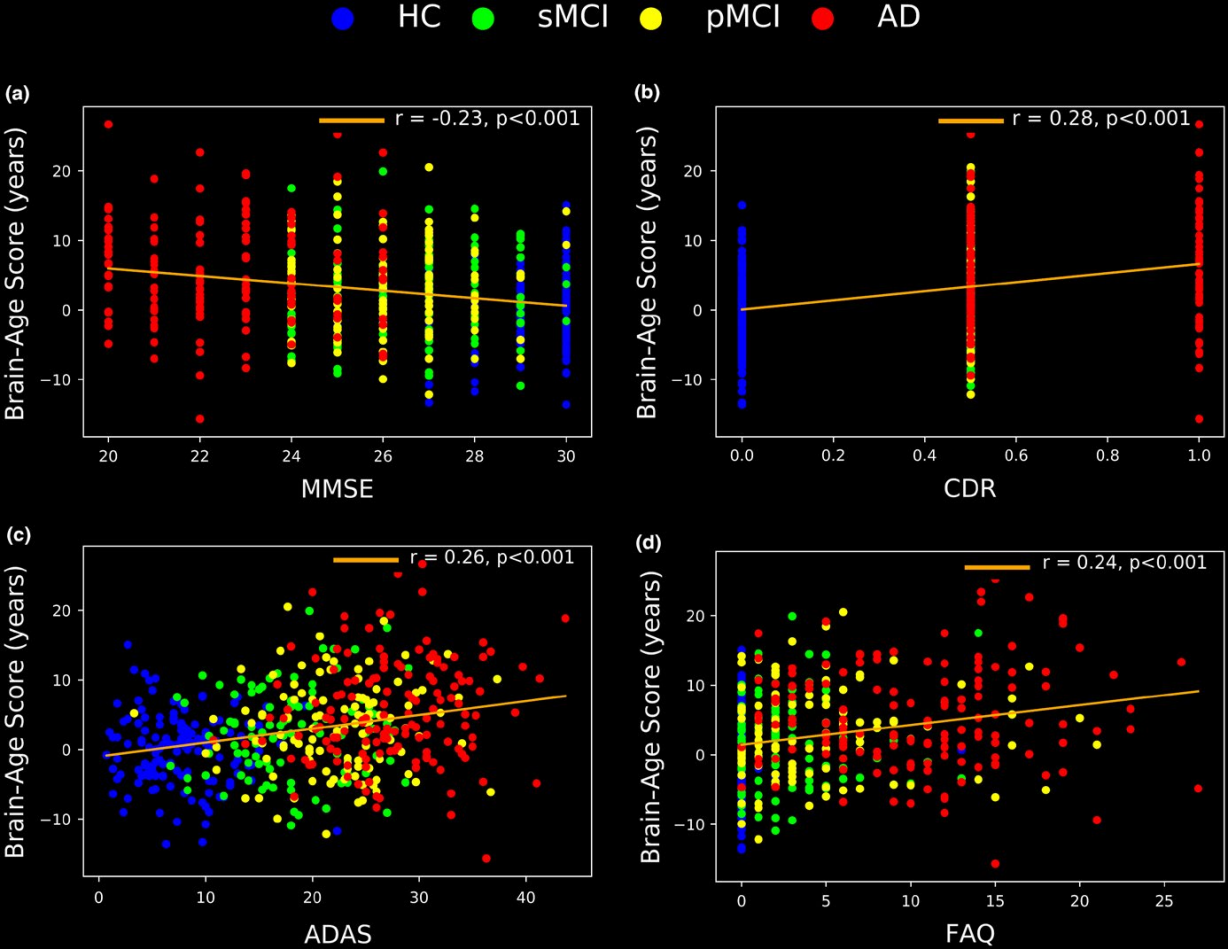
The correlations between the BAS results and the neuropsychological scores in the evaluation group: (a) MMSE, (b) CDR, (c) ADAS, and (d) FAQ. The neuropsychological scores were acquired at the baseline

**Figure 8:**
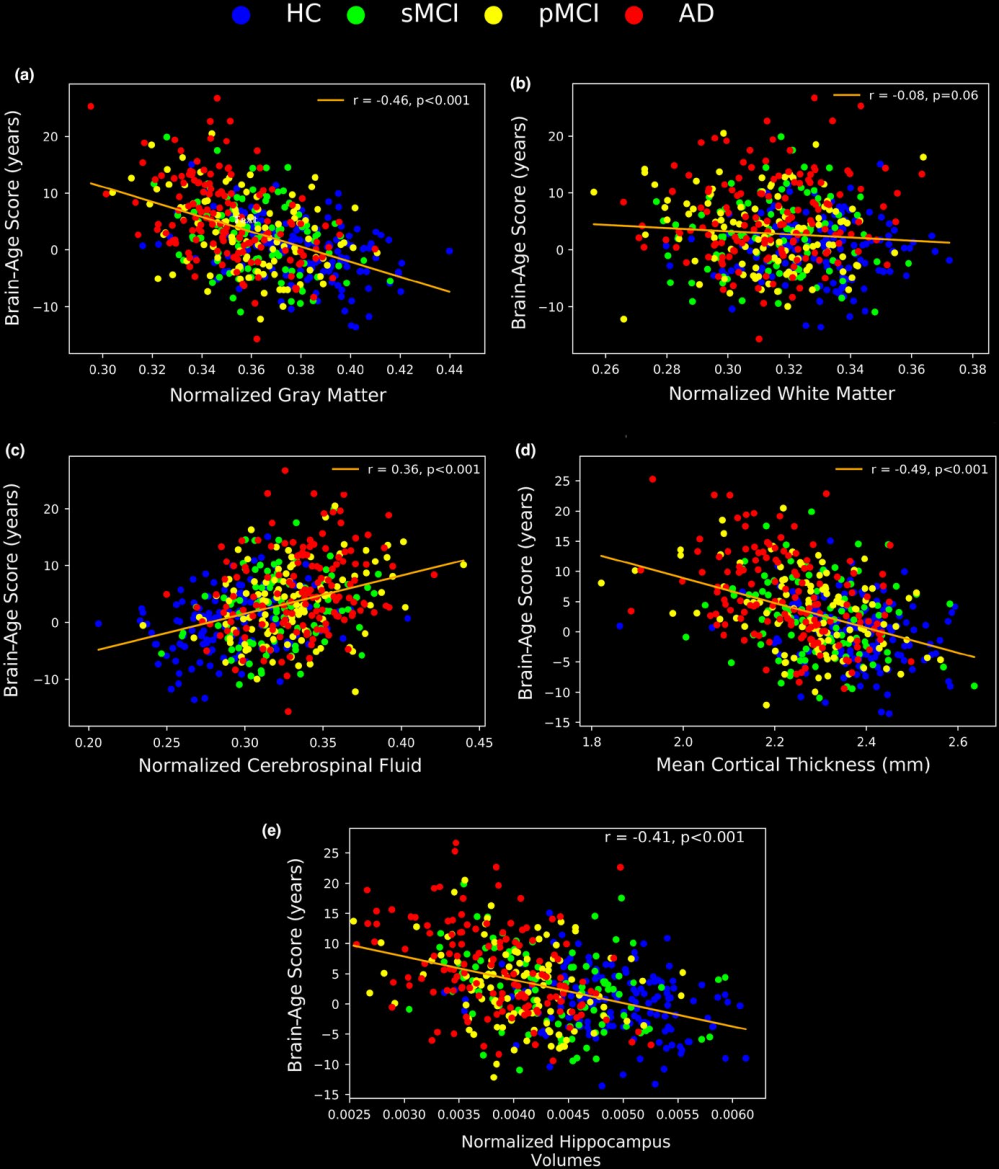
The correlations between the BAS results and the anatomical MRI measurements in the evaluation group: (a) nGM, (b) nWM, (c) nCSF, (d) mean cortical thickness, and (e) normalized hippocampus volume. The anatomical MRI measurements were acquired at the baseline

The associations between the BAS results and the neuropsychological scores as well as the associations between the BAS results and anatomical MRI measurements are illustrated in Figures 7 and 8, respectively. According to the correlation test results, the BAS has relationships with the MMSE, the CDR, the ADSR, the FAQ scores, the nGM, the nCSF, the mean cortical thickness, and the normalized hippocampus volume (*r* = −0.23, *r* = 0.28, *r* = 0.26, *r* = 0.24, *r* = −0.46, *r* = 0.36, *r* = −0.49 and *r* = −0.41, respectively, *p *<* *0.001). There was no significant correlation between the BAS and the nWM.

## 6 DISCUSSION

We conducted the present empirical study to develop an automated framework for estimating the neuroanatomical age in individuals with AD or MCI and healthy subjects, and to determine the utility of our index (the BAS) as a computerized AD screening score. The BAS of the subjects is determined by subtracting the subject's neuroanatomical age from his or her chronological age. The results of our present analyses demonstrated that the difference between the neuroanatomical age and chronological age increased along with the level of AD; the mean BAS values were +0.07, +2.38, +3.15, and +5.36 years for our HCs, sMCI, pMCI, and AD subjects, respectively. A higher positive BAS implies higher accelerated/precocious brain aging, with an increasing BAS reflecting increasing severity of the level of AD.

Consequently, the mean BAS values of +5.63 and +3.15 for our present AD and pMCI groups indicate that the accelerated brain aging among AD and pMCI patients is higher than that of the sMCI and HC subjects with the mean BAS values of +2.38 and +0.07, respectively. Our statistical analysis showed a greater elevation of predicted age from chronological age in younger AD patients than older AD patients. This finding is in agreement with other studies that demonstrated a faster rate of AD progression in early‐onset than in late‐onset AD patients (Koss et al., 1996).

In several recent studies, the authors used the PCA method to reduce the high‐dimensional input space into a low‐dimensional space (Franke, Hagemann, Schleussner, & Gaser, 2015; Franke et al., 2010, 2012, 2017). In the PCA method, the number of principal components has a major effect on the performance, and it is usually determined manually. The effect of PCA data reduction and the influence of different principal components on the accuracy of an age estimation model have been addressed (Franke et al., 2010). One advantage of our proposed brain‐age framework is the introduction of a novel and fully automated feature selection approach based on feature‐ranking for brain‐age estimations. The proposed feature selection method not only introduces data reduction but also promotes the framework's performance by decreasing the MAE.

We also assessed the association between the obtained BAS values and the neuropsychological scores as well as between the BAS results and anatomical MRI measurements. The correction afforded by our proposed brain‐age approach confirms the results of the traditional screening tools (i.e., the MMSE, CDR, ADAS, and FAQ) and anatomical MRI measurements (i.e., nGM, nCSF, mean cortical thickness, and hippocampus atrophy) for the diagnosis of AD. The significant correlation (*p *<* *0.001) between the BAS and the two general cognitive assessments in AD (the MMSE and CDR) implies that the BAS has potential as a fully automated criterion that can be used to assess the level of brain atrophy in AD.

The main advantage of our proposed brain‐age approach is that it is a fully automated approach at all stages (from preprocessing, feature selection, and training a regression model), which functions based on only an MRI scan of the brain. Our approach summarizes a pattern across the whole brain to one single value (the BAS). In contrast, common approaches in the diagnosis of AD (e.g., the MMSE, CDR, ADAS and FAQ) focus on self‐reported results of a survey, which can be influenced by common method bias.

One limitation of our study is that the proposed approach using the BAS could not distinguish the sMCI patients from the pMCI patients (mean difference [MD] = −0.77, *p *=* *0.81). The reason for this may be due to the similar patterns shown by sMCI and pMCI patients at the baseline. For example, there were no significant differences between the sMCI and pMCI patients in terms of the MMSE (MD = 0.47, *p *=* *0.12) and CDR (MD = 0, *p *=* *1.00) scores, which are widely used in AD studies. This is why the prediction of MCI‐to‐AD conversion is the one of the most difficult tasks in AD studies. In future research, we plan to use the cortical thickness and diffusion properties (Irimia, Torgerson, Goh, & Van Horn, 2015) and to combine MRI features with nonimaging characteristics (DeCarlo, Tuokko, Williams, Dixon, & MacDonald, 2014), not only to address this limitation but also to further develop the brain‐age technique toward the goal of revealing the earliest indications of AD in symptomatic and asymptomatic individuals. Several studies have successfully investigated multimodal data such as MRI, PET for developing a high accurate AD classification or MCI conversion prediction frameworks (Ortiz, Munilla, Álvarez‐Illán, Górriz, & Ramírez, 2015; Zhang & Shen, 2012; Zhang, Wang, Zhou, Yuan, & Shen, 2011). Another direction for future study may be to use multimodal data (i.e., MRI and PET) to present a high accurate and robust brain‐age model.

## 7 CONCLUSION

We developed a fully automated framework (including preprocessing, feature selection, and training a regression model) for estimating the neurological age in AD, MCI, and healthy individuals, using only a standard MRI scan. The proposed framework was trained using 385 healthy subjects and evaluated in a case study consisting of 146 healthy controls, 102 patients with sMCI, 112 pMCI patients, and 147 individuals with AD from the J‐ADNI database, considering their baseline information. We evaluated the efficacy of the proposed framework by performing correlation tests between the BAS and traditional screening tools of AD as well as between the BAS and anatomical MRI measurements. Ultimately, our findings indicate that the BAS has the potential to be a robust, reliable, and computerized biomarker for the diagnosis of the level of AD as well as other neuropsychological conditions.

## APPENDIX 1

### 1.1

The J‐ADNI was a multicenter study assessing neuroimaging in diagnosis and longitudinal monitoring that was started in 2008 in Japan by the New Energy and Industrial Technology Development Organization (NEDO) and the Ministry of Health, Labour and Welfare (MHLW). All of the participants were recruited at 38 Japanese clinical sites. They were followed up for 2–3 years using 1.5‐T MRI, positron emission tomography (PET), biological fluid analysis, and neuropsychological batteries. All of the protocols were designed to be as compatible as possible to those of the ADNI. For additional details about the J‐ADNI, see the previous article by the J‐ADNI (Iwatsubo, 2010). Participants were 60 to 84 years of age, generally healthy, spoke Japanese, lived at home, and had a study partner. Details of the J‐ADNI inclusion and exclusion criteria can be found at https://upload.umin.ac.jp/cgi-open-bin/ctr/ctr.cgi?function=brows&action=brows&recptno=R000001668&type=summary&language=E. Briefly, the inclusion criteria for cognitively normal (CN) participants included the following: a score of 24–30 on the Mini‐Mental State Examination (MMSE) (Folstein et al., 1975), Japanese version; a global score of 0 on the CDR, Japanese version; and an education‐adjusted score above the cutoff level on the Wechsler Memory Scale‐Revised (WMS‐R) Logical Memory II (Wechsler & Stone, 1987), Japanese version (education for 0–9 years was ≥3, for 10–15 years was ≥5, and for >15 years was ≥9). The inclusion criteria for the MCI subjects were a score of 24–30 on the MMSE, memory disturbance identified by the study partner with or without the subjective complaint of the participant, a score of 0.5 on the CDR, and an education‐adjusted score below the cutoff level on the WMS‐R Logical Memory II (education for 0–9 years was ≤2, for 10–15 years was ≤4, and for >15 years was ≤8). The inclusion criteria for AD subjects was a score of 20–26 on the MMSE score, a score of 0.5 or 1 on the CDR, and an education‐adjusted score below the cutoff level on the WMS‐R Logical Memory II (same as for MCI). AD subjects also had to meet the criteria of the NINCDS‐ADRDA (the National Institute of Neurological and Communicative Diseases and Stroke and the Alzheimer's Disease and Related Disorders Association) for probable AD (McKhann et al., 1984). Exclusion criteria included brain lesions on screening or baseline MRI, neurological and psychiatric disorders other than AD, addiction to alcohol or other drugs, and use of psychoactive drugs or warfarin.

The institutional review boards at all participating sites approved the data collection procedures and written informed consent was obtained from all participants. If participants were not capable of agreeing, their study partner signed the informed consent form in substitution. A total of 750 participants were first recruited at the 38 clinical sites in Japan. Those who provided written informed consent and passed screening based on the above inclusion/exclusion criteria were enrolled in the J‐ADNI study. Finally, 537 participants were enrolled. The 537 participants underwent brain MRI at baseline. Follow‐up MRI was performed at 6, 12, and 24 months for all participants and at 36 months only for MCI and CN participants. MCI participants additionally underwent MRI at 18 months. Clinical and cognitive assessments were also performed for all participants at the time of the baseline and follow‐up scans. These assessments included MMSE, ADAS‐Cog, and CDR‐SB. Data were used for analysis from 146 AD, 102 sMCI, 112 pMCI and 147 NC participants.

## References

[brb31020-bib-0001] Bedell, B. J. , Carbonell, F. , Charil, A. , Zijdenbos, A. P. , Evans, A. C. , Sevigny, J. , & Chiao, P. (2014). Optimized, fully‐automated classification of amyloid‐positive subjects based on [18f] florbetapir PET scans. Alzheimer's Dement. J. Alzheimer's Assoc., 10, P708–P709. 10.1016/j.jalz.2014.05.1303

[brb31020-bib-0002] Beheshti, I. , & Demirel, H. (2016). Feature‐ranking‐based Alzheimer's disease classification from structural MRI. Magnetic Resonance Imaging, 34, 252–263. 10.1016/j.mri.2015.11.009 26657976

[brb31020-bib-0003] Beheshti, I. , Demirel, H. , Farokhian, F. , Yang, C. , & Matsuda, H. (2016). Structural MRI‐based detection of Alzheimer's disease using feature ranking and classification error. Computer Methods and Programs in Biomedicine, 137, 177–193. 10.1016/j.cmpb.2016.09.019 28110723

[brb31020-bib-0004] Beheshti, I. , Olya, H. G. T. , & Demirel, H. (2016). Risk assessment of alzheimer's disease using the information diffusion model from structural magnetic resonance imaging. Journal of Alzheimer's Disease, 52, 1–8. 10.3233/JAD-151176 27060960

[brb31020-bib-0005] Chaves, R. , Ramírez, J. , Górriz, J. M. , & Puntonet, C. G. (2012). Association rule‐based feature selection method for Alzheimer's disease diagnosis. Expert Systems With Applications, 39, 11766–11774. 10.1016/j.eswa.2012.04.075

[brb31020-bib-0006] Cole, J. H. (2017). Neuroimaging‐derived brain‐age: An ageing biomarker? Aging (Albany, NY), 9, 1861–1862. 10.18632/aging.101286 28858849 PMC5611979

[brb31020-bib-0007] Cole, J. H. , Leech, R. , & Sharp, D. J. (2015). Prediction of brain age suggests accelerated atrophy after traumatic brain injury. Annals of Neurology, 77, 571–581. 10.1002/ana.24367 25623048 PMC4403966

[brb31020-bib-0008] Cole, J. H. , Underwood, J. , Caan, M. W. A. , De Francesco, D. , Van Zoest, R. A. , Leech, R. , … Sharp, D. (2017). Increased brain‐predicted aging in treated HIV disease. Neurology, 88, 1349–1357. 10.1212/WNL.0000000000003790 28258081 PMC5379929

[brb31020-bib-0009] Cuingnet, R. , Gerardin, E. , Tessieras, J. , Auzias, G. , Lehéricy, S. , Habert, M. O. , … Colliot, O. (2011). Automatic classification of patients with Alzheimer's disease from structural MRI: A comparison of ten methods using the ADNI database. NeuroImage, 56, 766–781. 10.1016/j.neuroimage.2010.06.013 20542124

[brb31020-bib-0010] DeCarlo, C. A. , Tuokko, H. A. , Williams, D. , Dixon, R. A. , & MacDonald, S. W. S. (2014). BioAge: Toward a multi‐determined, mechanistic account of cognitive aging. Ageing Research Reviews, 18, 95–105. 10.1016/j.arr.2014.09.003 25278166 PMC4258131

[brb31020-bib-0011] Dosenbach, N. U. F. , Nardos, B. , Cohen, A. L. , Fair, D. A , Power, D. , Church, J. A , … Schlaggar, B. L. (2011). Prediction of individua brain maturity using fMRI. Science (80‐.), 329, 1358–1361. 10.1126/science.1194144.prediction PMC3135376 20829489

[brb31020-bib-0012] Duchesne, S. , & Gravel, P. (2016). Estimating brain age across the life span using MRI appearance. Alzheimer's & Dementia, 12, P111 10.1016/j.jalz.2016.06.180

[brb31020-bib-0013] Erus, G. , Battapady, H. , Satterthwaite, T. D. , Hakonarson, H. , Gur, R. E. , Davatzikos, C. , … Gur, R. C. (2015). Imaging patterns of brain development and their relationship to cognition. Cerebral Cortex, 25, 1676–1684. 10.1093/cercor/bht425 24421175 PMC4428302

[brb31020-bib-0014] Fillenbaum, G. G. , & Smyer, M. A. (1981). The development, validity, and reliability of the OARS multidimensional functional assessment questionnaire. Journal of Gerontology, 36, 428–434. 10.1093/geronj/36.4.428 7252074

[brb31020-bib-0015] Folstein, M. F. , Folstein, S. E. , & McHugh, P. R. (1975). “Mini‐mental state”. A practical method for grading the cognitive state of patients for the clinician. Journal of Psychiatric Research, 12, 189–198. https://doi.org/0022-3956(75)90026-6[pii] 1202204 10.1016/0022-3956(75)90026-6

[brb31020-bib-0016] Franke, K. , Clarke, G. D. , Dahnke, R. , Gaser, C. , Kuo, A. H. , Li, C. , … Nathanielsz, P. W. (2017). Premature brain aging in baboons resulting from moderate fetal undernutrition. Frontiers in Aging Neuroscience, 9, 10.3389/fnagi.2017.00092 PMC5386978 28443017

[brb31020-bib-0017] Franke, K. , & Gaser, C. (2012). Longitudinal changes in individual *BrainAGE* in healthy aging, mild cognitive impairment, and alzheimer's disease 1. Data used in preparation of this article were obtained from the Alzheimer's Disease Neuroimaging Initiative (ADNI) database (adni.loni). GeroPsych (Bern), 25, 235–245. 10.1024/1662-9647/a000074

[brb31020-bib-0018] Franke, K. , & Gaser, C. (2014). Dementia classification based on brain age estimation. Proc MICCAI Workshop Challenge on Computer‐Aided Diagnosis of Dementia Based on Structural MRI Data.

[brb31020-bib-0019] Franke, K. , Hagemann, G. , Schleussner, E. , & Gaser, C. (2015). Changes of individual BrainAGE during the course of the menstrual cycle. NeuroImage, 115, 1–6. 10.1016/j.neuroimage.2015.04.036 25913700

[brb31020-bib-0020] Franke, K. , Luders, E. , May, A. , Wilke, M. , & Gaser, C. (2012). Brain maturation: Predicting individual Brain AGE in children and adolescents using structural MRI. NeuroImage, 63, 1305–1312. 10.1016/j.neuroimage.2012.08.001 22902922

[brb31020-bib-0021] Franke, K. , Ziegler, G. , Klöppel, S. , & Gaser, C. (2010). Estimating the age of healthy subjects from T1‐weighted MRI scans using kernel methods: Exploring the influence of various parameters. NeuroImage, 50, 883–892. 10.1016/j.neuroimage.2010.01.005 20070949

[brb31020-bib-0022] Fujishima, M. , Kawaguchi, A. , Maikusa, N. , Kuwano, R. , Iwatsubo, T. , & Matsuda, H. (2017). Sample size estimation for alzheimer's disease trials from Japanese ADNI serial magnetic resonance imaging. Journal of Alzheimer's Disease, 56, 75–88. 10.3233/JAD-160621 PMC5240548 27911297

[brb31020-bib-0023] Gaser, C. , Franke, K. , Klöppel, S. , Koutsouleris, N. , & Sauer, H. (2013). BrainAGE in mild cognitive impaired patients: Predicting the conversion to alzheimer's disease. PLoS ONE, 8, 10.1371/journal.pone.0067346 PMC3695013 23826273

[brb31020-bib-0024] Irimia, A. , Torgerson, C. M. , Goh, S. Y. M. , & Van Horn, J. D. (2015). Statistical estimation of physiological brain age as a descriptor of senescence rate during adulthood. Brain Imaging and Behavior, 9, 678–689. 10.1007/s11682-014-9321-0 25376330 PMC4424195

[brb31020-bib-0025] Iwatsubo, T. (2010). Japanese alzheimer's disease neuroimaging Initiative: Present status and future. Alzheimer's & Dementia, 6, 297–299. 10.1016/j.jalz.2010.03.011 20451880

[brb31020-bib-0026] Khedher, L. , Ramírez, J. , Górriz, J. M. , Brahim, A. , & Segovia, F. (2015). Early diagnosis of Alzheimer׳s disease based on partial least squares, principal component analysis and support vector machine using segmented MRI images. Neurocomputing, 151, 139–150. 10.1016/j.neucom.2014.09.072

[brb31020-bib-0027] Klöppel, S. , Stonnington, C. M. , Chu, C. , Draganski, B. , Scahill, R. I. , Rohrer, J. D. , … Frackowiak, R. S. (2008). Automatic classification of MR scans in Alzheimer's disease. Brain, 131, 681–689. 10.1093/brain/awm319 18202106 PMC2579744

[brb31020-bib-0028] Koss, E. , Edland, S. , Fillenbaum, G. , Mohs, R. , Clark, C. , Galasko, D. , … Morris, J. C. (1996). Clinical and neuropsychological differences between patients with earlier and later onset of Alzheimer's disease A CERAD analysis, part XII. Neurology, 46, 136–141. 10.1212/WNL.46.1.136 8559362

[brb31020-bib-0029] Koutsouleris, N. , Davatzikos, C. , Borgwardt, S. , Gaser, C. , Bottlender, R. , Frodl, T. , … Meisenzahl, E. (2014). Accelerated brain aging in schizophrenia and beyond: A neuroanatomical marker of psychiatric disorders. Schizophrenia Bulletin, 40, 1140–1153. 10.1093/schbul/sbt142 24126515 PMC4133663

[brb31020-bib-0030] Lancaster, J. , Lorenz, R. , Leech, R. , Cole, J. H. , Jena, F. , & Cole, J. H. (2018). Bayesian optimization for neuroimaging pre‐processing in brain age classification and prediction. Frontiers in Aging Neuroscience, 10, 1–10. 10.3389/fnagi.2018.00028 29483870 PMC5816033

[brb31020-bib-0031] Lin, L. , Jin, C. , Fu, Z. , Zhang, B. , Bin, G. , & Wu, S. (2016). Predicting healthy older adult's brain age based on structural connectivity networks using artificial neural networks. Computer Methods and Programs in Biomedicine, 125, 8–17. 10.1016/j.cmpb.2015.11.012 26718834

[brb31020-bib-0032] Liu, M. , Zhang, D. , & Shen, D. (2016). Relationship induced multi‐template learning for diagnosis of alzheimer's disease and mild cognitive impairment. IEEE Transactions on Medical Imaging, 35, 1463–1474. 10.1109/TMI.2016.2515021 26742127 PMC5572669

[brb31020-bib-0033] López, M. , Ram'irez, J. , Górriz, J. M. , Álvarez, I. , Salas‐Gonzalez, D. , Segovia, F. , & Puntonet, C. G. (2009). Computer aided diagnosis of alzheimer's disease using principal component analysis and bayesian classifiers. In The Sixth International Symposium on Neural Networks (ISNN 2009), 213–221.

[brb31020-bib-0034] Luders, E. , Cherbuin, N. , & Gaser, C. (2016). Estimating brain age using high‐resolution pattern recognition: Younger brains in long‐term meditation practitioners. NeuroImage, 134, 508–513. 10.1016/j.neuroimage.2016.04.007 27079530

[brb31020-bib-0035] Martinez‐Murcia, F. J. , Górriz, J. M. , Ram'irez, J. , Ortiz, A. ; For The Alzheimer's Disease Neuroimaging Initiative . (2016). A spherical brain mapping of MR images for the detection of Alzheimer's disease. Current Alzheimer Research, 13, 575–588. 10.2174/1567205013666160314145158 26971941

[brb31020-bib-0036] McKhann, G. , Drachman, D. , Folstein, M. , Katzman, R. , Price, D. , & Stadlan, E. M. (1984). Clinical diagnosis of Alzheimer's disease report of the NINCDS‐ADRDA work group under the auspices of department of health and human services task force on Alzheimer's isease. Neurology, 34, 939 10.1212/WNL.34.7.939 6610841

[brb31020-bib-0037] Mikhno, A. , Redei, J. , Mann, J. , & Parsey, R. (2015). Accurate early Alzheimer's disease detection: Cross‐tracer validation of automated voxel‐based amyloid PET SUVR in independent datasets. Alzheimer's & Dementia, 11, P880 10.1016/j.jalz.2015.08.086

[brb31020-bib-0038] Mohs, R. C. , Rosen, W. G. , & Davis, K. L. (1983). The Alzheimer's disease assessment scale: An instrument for assessing treatment efficacy. Psychopharmacology Bulletin, 19, 448. 6635122

[brb31020-bib-0039] Moradi, E. , Hallikainen, I. , Hänninen, T. , Tohka, J. , & Neuroimaging, D. (2017). Rey's Auditory Verbal Learning Test scores can be predicted from whole brain MRI in Alzheimer's disease NeuroImage : Clinical. NeuroImage Clinical, 13, 415–427. 10.1016/j.nicl.2016.12.011 28116234 PMC5233798

[brb31020-bib-0040] Morris, J. C. (1993). The Clinical Dementia Rating (CDR): Current version and scoring rules. Neurology, 43(11), 2412–2414. 10.1212/WNL.43.11.2412-a 8232972

[brb31020-bib-0041] Ortiz, A. , Munilla, J. , Álvarez‐Illán, I. , Górriz, J. M. , Ramírez, J. ; Alzheimer's Disease Neuroimaging Initiative (2015). Exploratory graphical models of functional and structural connectivity patterns for Alzheimer's Disease diagnosis. Frontiers in Computational Neuroscience, 9, 132. 26578945 10.3389/fncom.2015.00132 PMC4630314

[brb31020-bib-0042] Presser, S. , Couper, M. P. , Lessler, J. T. , Martin, E. , Rothgeb, J. M. , & Singer, E. (2004). Methods for testing and evaluating survey questions. Public Opinion, 68, 109–130. 10.1093/poq

[brb31020-bib-0043] Ramírez, J. , Górriz, J. M. , Segovia, F. , Chaves, R. , Salas‐Gonzalez, D. , López, M. , … Padilla, P. (2010). Computer aided diagnosis system for the Alzheimer's disease based on partial least squares and random forest SPECT image classification. Neuroscience Letters, 472, 99–103. 10.1016/j.neulet.2010.01.056 20117177

[brb31020-bib-0044] Scholkopf, B. , Sung, K.‐K. , Burges, C. J. C. , Girosi, F. , Niyogi, P. , Poggio, T. , & Vapnik, V. (1997). Comparing support vector machines with Gaussian kernels to radial basis function classifiers. IEEE Transactions on Signal Processing, 45, 2758–2765. 10.1109/78.650102

[brb31020-bib-0045] Segovia, F. , Górriz, J. M. , Ramírez, J. , Salas‐González, D. , & Álvarez, I. (2013). Early diagnosis of Alzheimer's disease based on partial least squares and support vector machine. Expert Systems with Applications, 40, 677–683. 10.1016/j.eswa.2012.07.071

[brb31020-bib-0046] Shen, Q. , Loewenstein, D. A. , Potter, E. , Zhao, W. , Appel, J. , Greig, M. T. , … Duara, R. (2011). Volumetric and visual rating of magnetic resonance imaging scans in the diagnosis of amnestic mild cognitive impairment and Alzheimer's disease. Alzheimer's & Dementia, 7, e101–e108. 10.1016/j.jalz.2010.07.002 PMC3145968 21784342

[brb31020-bib-0047] Smola, A. J. , & Schölkopf, B. (2004). A tutorial on support vector regression. Statistics and Computing, 14, 199–222. 10.1023/B:STCO.0000035301.49549.88

[brb31020-bib-0048] Stonnington, C. M. , Chu, C. , Klöppel, S. , Jack, C. R. , Ashburner, J. , & Frackowiak, R. S. J. (2010). Predicting clinical scores from magnetic resonance scans in Alzheimer's disease. NeuroImage, 51, 1405–1413. 10.1016/j.neuroimage.2010.03.051 20347044 PMC2871976

[brb31020-bib-0049] Vapnik, V. (2013). The nature of statistical learning theory. New York, NY, USA: Springer science & business media.

[brb31020-bib-0050] Wang, J. , Dai, D. , Li, M. , Hua, J. , & He, H. (2012). Human age estimation with surface‐based features from MRI images. In International Workshop on Machine Learning in Medical Imaging, 111–118.

[brb31020-bib-0051] Wechsler, D. , & Stone, C. P. (1987). Wechsler memory scale‐revised. San Antonio: Psychological Corporation.

[brb31020-bib-0052] Welling, M. (2004). Support vector regression Toronto. Toronto: Department of Computer Science University.

[brb31020-bib-0053] Zhang, D. , & Shen, D. (2012). Multi‐modal multi‐task learning for joint prediction of multiple regression and classification variables in Alzheimer's disease. NeuroImage, 59, 895–907. 10.1016/j.neuroimage.2011.09.069 21992749 PMC3230721

[brb31020-bib-0054] Zhang, D. , Wang, Y. , Zhou, L. , Yuan, H. , Shen, D. ; Alzheimer's Disease Neuroimaging Initiative (2011). Multimodal classification of Alzheimer's disease and mild cognitive impairment. NeuroImage, 55, 856–867. 10.1016/j.neuroimage.2011.01.008 21236349 PMC3057360

[brb31020-bib-0055] Zhang, J. , Zheng, C.‐H. , Xia, Y. , Wang, B. , & Chen, P. (2017). Optimization enhanced genetic algorithm‐support vector regression for the prediction of compound retention indices in gas chromatography. Neurocomputing, 240, 183–190. 10.1016/j.neucom.2016.11.070

[brb31020-bib-0056] Zhou, N. , & Wang, L. (2007). A modified T‐test feature selection method and its application on the hapmap genotype data. Genomics, Proteomics & Bioinformatics, 5, 242–249. 10.1016/S1672-0229(08)60011-X PMC5054219 18267305

